# Construction of a Novel Damage-Associated Molecular-Pattern-Related Signature to Assess Lung Adenocarcinoma’s Prognosis and Immune Landscape

**DOI:** 10.3390/biom14010108

**Published:** 2024-01-15

**Authors:** Xinyue Liu, Shuxi Yao, Yanqi Feng, Piao Li, Yiming Li, Shu Xia

**Affiliations:** Department of Oncology, Tongji Hospital, Tongji Medical College, Huazhong University of Science and Technology, Wuhan 430030, China; liuxinyue01@hust.edu.cn (X.L.); m202276300@hust.edu.cn (S.Y.); d202382250@hust.edu.cn (Y.F.); m202276270@hust.edu.cn (P.L.); d202282140@hust.edu.cn (Y.L.)

**Keywords:** damage-associated molecular patterns, immunogenic death, immunotherapy, PANX1

## Abstract

Immunogenic death (ICD) stimulates adaptive immunity and affects immunotherapeutic efficacy, an important part of which is damage-associated molecular patterns (DAMPs). However, the function of these DAMPs for lung adenocarcinoma (LUAD) remains obscure. We initially found differentially expressed genes (DEGs) with prognostic significance related to DAMPs with the TCGA database and then used the least absolute shrinkage and selection operator (LASSO) regression to create a risk signature strongly correlated with overall survival (OS) with eight DEGs. Validation was performed externally using the external data set GSE68465. Lower-risk LUAD patients were found to be more chemotherapy-resistant and enriched for more immune-related pathways than those with higher risk scores, and patients with different risks showed different levels of immune cell infiltration. *PANX1*, a crucial gene closely associated with lung adenocarcinoma, was identified using the weighted correlation network analysis (WGCNA), and experiments revealed that PANX1 promotes the proliferation as well as invasion of LUAD cells. Furthermore, PANX1 was found to be positively correlated with CD274, CD276, and M2 macrophage markers. We developed and validated an entirely new gene signature related to DAMPs that may be useful for LUAD patient prognosis, immune microenvironment, and chemotherapeutic drug sensitivity prediction. The results may also guide clinical immunotherapy and chemotherapy approaches for LUAD patients.

## 1. Introduction

With a 5-year survival rate of 10% to 20% for patients diagnosed between 2010 and 2014 in the majority of nations, lung cancer continues to be the most prevalent cause of mortality due to cancer [1]. Moreover, in the context of cancer of the lungs, lung adenocarcinoma (LUAD) is the most prevalent subtype of non-small cell lung cancer (NSCLC), which is the most prevalent type of lung cancer overall [2]. LUAD is a highly heterogeneous malignancy whose treatment options, including surgery, are highly dependent on the disease’s stages, molecular tumor markers, and other factors, and for LUAD patients, immunotherapy represents a breakthrough in recent stages [3]. Despite the fact that immune checkpoint inhibitors (ICBs) have shown some degree of success in treatment, there is still some uncertainty about their efficacy, and only a few patients have shown favorable effective efficacy to these medications up to this time [4]. The assessment of patient prognosis and the prediction of treatment regimen feasibility, as well as how medication resistance may be overcome, remain major roadblocks in the fight against lung cancer.

Damage-associated molecular patterns (DAMPs) are diverse, and a portion of them are released extracellularly following cell death or damage, eliciting primarily inflammatory and immune system responses. In addition, it has been demonstrated that a subset of DAMPs is generated during apoptosis in some instances, which induces immunogenic death (ICD) and can stimulate tumor immune responses by promoting antigen presentation and T cell activation in the immune system. This study focused on such DAMPs that have been reported to be linked to ICD. The release of DAMPs is a defining feature of ICD, which is a form of regulated immune mortality [5,6]. Furthermore, the identification and characterization of DAMPs have provided valuable insights into the mechanisms underlying the immune response to tumors. Specifically, DAMPs activate and promote the immunogenicity of antigen-presenting cells (APCs) and regulate the recruitment, activation, and maturation of T cells by interacting with their associated ligands, which are essential to the antitumor process. One class of DAMPs, high-mobility group box 1, is known to be secreted extracellularly and to bind to Toll-like receptor 4 to stimulate maturation and antigen presentation in APCs, in turn activating CTLs [7,8]. Similarly, adenosine-5′-triphosphate (ATP) is released and then binds to its ligand P2X7 to stimulate APC maturation and release cytokines [9,10]. Calreticulin (CALR) is another kind of DAMP that can bind to CD91 [11], promoting the DC maturation and phagocytosis of dying tumor cells [12].

Multiple studies have shown that ICD induced by DAMPs has significant clinical relevance as an immunotherapeutic tool. Specifically, it has been reported that tumor progression is efficiently inhibited by using ICD inducers in conjunction with immune checkpoint inhibitors [13,14]. This approach takes advantage of the immunostimulatory properties of DAMPs to enhance the antitumor immune response. Although the importance of ICD-related DAMPs and their related pattern-recognition receptors in tumor immunity has been explored, there are few studies evaluating their gene characteristics for predicting the prognosis, drug sensitivity, and immune profiling of LUAD patients.

**Figure 1:**
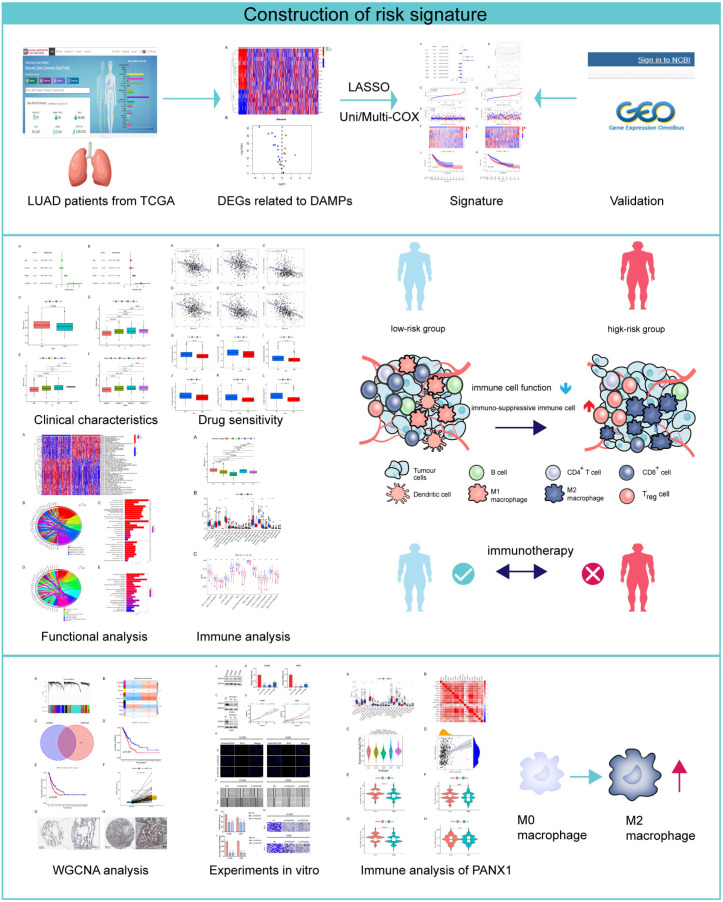
Flowchart of this study.

In this study, we aimed to develop a prognostic risk signature using DAMP-related genes. To achieve this, we started by extracting gene expression data from the TCGA database and identified differentially expressed DAMP-related genes. Using Cox analysis and LASSO regression, we established a robust eight-gene risk signature that could effectively predict clinical outcomes. Furthermore, we evaluated the predictive value of this risk model not only for clinical outcomes but also for drug sensitivity and the immune landscape. The signature was validated using data from the GEO database, ensuring its reliability and applicability across different datasets. We also conducted WGCNA analysis and performed functional experiments specifically focusing on the key gene, *PANX1*. This comprehensive approach allowed us to uncover valuable insights into the prognostic and functional significance of DAMP-related genes in LUAD, highlighting their potential as biomarkers for LUAD patients (Figure 1, Supplementary Figure S1).

## 2. Materials and Methods

### 2.1. Processing of Downloaded Data

We accessed the TCGA database (https://portal.gdc.cancer.gov, accessed on 3 December 2022) to gather clinical and transcriptome data for LUAD. We processed transcriptomic data as transcripts per million (TPM) and filtered LUAD samples lacking survival statistics. To verify the accuracy of the results, we obtained the GSE68465 microarray dataset from the Gene Expression Omnibus (GEO) (https://www.ncbi.nlm.nih.gov/gds, accessed on 3 December 2022).

### 2.2. Screening of Genes with Differential Expression Associated with DAMPs

Thirty-two genes all related to DAMPs were summarized in the concluding standardized database based on a prior investigation (*CALR*, *HMGB1*, *HMGN1*, *IL1A*, *IL33*, *ROCK1*, *PANX1*, *BCL2*, *PPIA*, *HSPA4*, *HSP90AA1*, *TLR2*, *TLR3*, *TLR4*, *TLR7*, *TLR9*, *CLEC4E*, *CLEC7A*, *NLRP3*, *DDX58*, *IFIH1*, *CGAS*, *AIM2*, *AGER*, *TREM1*, *FPR1*, *FPR2*, *P2Y2R*, *P2Y6R*, *P2Y12R*, *CASR*, *P2RX7*) [15]. Expression profiles of genes linked to DAMPs were retrieved from TCGA database samples using the criterion |logFC| > 0.585 (difference multiplier > 1.5). This stage was carried out using the “limma” R package to obtain differentially expressed genes (DEGs) in tumor tissues when compared to normal tissues.

### 2.3. Assessment of a Risk Signature Based on DAMP-Associated Genes

The least absolute shrinkage and selection operator (LASSO) Cox regression analysis was used to identify the genes and regression coefficients with the greatest predictive power after screening for prognostic DEGs with univariate Cox analysis and, finally, to build a risk signature on the most promising 8 genes. Using the “survival” and “survminer” R packages, the prognostic value of the risk signature was evaluated. Using PCA as well as t-SNE analysis performed using the “Rtsne” R package, we evaluated the signature’s reliability, and the prognostic model was confirmed using the GEO-independent data set GSE68465.

### 2.4. Gene Mutation Analysis

The TCGA database was queried for LUAD mutation information, and the “maftools” package for R was used to generate visualizations of prognostic gene mutations together with co-mutation connections. The “spearman” approach was utilized to examine the *PANX1*-TMB correlation.

### 2.5. Comparative Analysis of Risk Signature and Clinical Characteristics

LUAD patients were subgrouped to assess the risk signature’s practicality based on age, T stage, N stage, and pathological TNM stage using the “limma” R package. To learn more about what variables contribute to overall survival in LUAD patients, we used the “survival” R package to perform the univariate as well as the multivariate Cox regression analysis of clinical characteristics and risk scores.

### 2.6. Development of a Prognostic Nomogram

A nomogram was established for LUAD patients to predict overall survival, which was executed via the “rms” and “survival” R packages. The clinical prognostic value of the nomogram was tested using calibration curves and univariate and multivariate Cox regression analyses.

### 2.7. Analysis of Tumor Immune Infiltration

The CIBERSORT algorithm is an indispensable instrument for precisely quantifying diverse immune cells, making it indispensable for a wide range of immunological research applications [16]. Using this instrument, we compared the differences in immune cell infiltration between groups at different risks. Risk scores were also calculated for six different immune subtypes. Furthermore, using the “ssgsea” method, we analyzed the relative immune function of the different groups. With the TISIDB database (http://cis.hku.hk/TISIDB/, accessed on 18 February 2023), we can query the association between any gene and immune trait [17]. We used this method to investigate whether or not PANX1 was linked to certain immune subtypes. The TIMER2.0 website (http://timer.cistrome.org/, accessed on 10 April 2023) provides coefficients for correlations between specific genes. Additionally, we used the “corrplot” R tool to map the correlation between *PANX1* and immune checkpoint-related genes.

### 2.8. Drug Sensitivity Analysis

The half-maximal inhibitory concentration (IC50) value is commonly employed for substance screening and evaluation. In this study, we compared the drug sensitivity performance between different risk groups using the drug-sensitivity-prediction algorithm in the “pRRophetic” R package to determine the IC50.

### 2.9. Immunotherapy Effectiveness

Immunophenotype scores (IPSs) are derived from gene-expression scores in the range of 0 to 10 that determine tumor immunogenicity, with IPS weighted positively for stimulatory factors and negatively for inhibitory factors, with higher scores correlating with higher immunogenicity. IPS is a good predictor of checkpoint blocker response [18], and we obtained information on this index from TCIA (https://tcia.at/home, accessed on 22 February 2023) for each LUAD patient.

### 2.10. Enrichment Analysis

One kind of technique for evaluating transcriptomic gene set enrichment is called gene set variation analysis (GSVA) [19]. To identify potential biological pathways and processes associated with “c2.cp.kegg.v7.4.symbols.gmt”, the “GSVA” R package was employed to enrich the gene set, and following the enrichment process, the results were analyzed and visualized. Furthermore, we employed the “limma” R package with the criteria that the log2|FC > 1| and the false discovery rate < 0.05 to discover DEGs between different risk groups. Furthermore, Gene Ontology (GO) alongside Kyoto Encyclopedia of Genes and Genomes (KEGG) enrichment analysis was conducted using the “clusterProfiler” R package, which allowed us to analyze the function of DEGs and their association with disease onset and progression in greater detail.

### 2.11. Weighted Gene Co-Expression Network Analysis

To identify co-expressed gene modules and core genes in the network, we analyzed the TCGA dataset utilizing the “WGCNA” R package, constructed the WGCNA network utilizing the unsigned topological matrix (TOM), and investigated the network modules and filtered the data using the “pickSoftThreshold” function. This allowed us to divide the data into modules, with each module containing characteristics that co-express similar genes.

### 2.12. Cell Transfection

At 37 °C and 5% CO_2_, we cultivated the normal human lung epithelial cell line BEAS-2B and the human LUAD cell lines H2228, H1299, and H358 from the cell bank at the Chinese Academy of Sciences. In view of the high expression of PANX1 in H358 and H1299 cell lines as shown by WB experiments, these two cell lines were selected as the subsequent transfected cell lines. Small interfering RNA (siRNA) was obtained from OBIO Technologies to target human *PANX1*. After culturing H358 and H1299 cells to 50% cell density, siRNA was mixed with reduced serum medium and transfection reagent from Invitrogen and incubated for 20 min, and the siRNA complex was added to fresh medium. Six hours after transfecting the cells, the medium was replaced. Following 24–48 h of transfection, cells were gathered for validation.

### 2.13. Quantitative Real-Time Fluorescence PCR

The cells were initially evenly distributed in 6-well plates. Once the cells reached 80% confluence, the medium was removed, and RNA was extracted using Takara’s Trizol reagent. A cDNA synthesis reaction solution was prepared with 1 μg of template RNA, 4 μL of 5× HisScript Ⅱ qRTSuperMix, and enzyme-free water reach to a total volume of 20 μL for each sample. The reaction solution was thoroughly mixed and subjected to reverse transcription at 50 °C for 15 min followed by 85 °C for 5 s to obtain the cDNA product. RT-qPCR amplification was performed using the cDNA as a template. The reaction mixture was prepared in 96-well plates, with 10 μL per well consisting of 2× ChamQ Universal SYBR qPCR Master Mix (5 μL), Forward Primer (0.2 μL), Reverse Primer (0.2 μL), cDNA (1 μL), and enzyme-free water (3.6 μL). Three replicate wells were set for each sample. The reaction conditions were as follows: initial denaturation at 95 °C for 30 s, followed by 40 cycles of denaturation at 95 °C for 10 s and annealing at 60 °C for 30 s, and a final extension step at 95 °C for 15 s, 60 °C for 60 s, and 95 °C for 15 s. Reagents for cDNA synthesis and RT-qPCR were purchased from Vazyme Biotech Co., Ltd. (Nanjing, China).

The primers for *PANX1* were 5′-CCATGGCCATCGCTCAACT-3′ and 5′-CTGTGTACCAATCGAGATCTCCTG-3′; the *GAPDH* primers were 5′-TGACTTCAACAGCGACCCA-3′ and 5′-CACCCTGTTGCTGTAGCCAAA-3′. We utilized the 2^−∆∆Ct^ method to ascertain the relative gene expression levels, using the *GAPDH* expression level as an internal standard. Relative gene expression levels were determined using 2^−∆∆Ct^ values, with *GAPDH* expression serving as an internal reference.

### 2.14. Western Blot (WB) Analysis

To assess the expression levels of PANX1 in LUAD cell lines (H1299, 358, H2228) and a normal lung epithelial cell line (BEAS-2B) and to confirm the transfection efficiency of si-*PANX1* in H1299 and H358 cells, Western blot experiments were conducted. Upon reaching an appropriate cell density in the culture dish, RIPA cell lysis buffer (100:1:1) supplemented with pre-added protease and phosphatase inhibitors was used for cell lysis on ice for 20–30 min, followed by centrifugation to collect the supernatant. The protein concentration was determined using the BCA method, and the calculated protein concentration was based on the measured OD value. Subsequently, 5× protein loading buffer was added, and the mixture was heated to 100 °C for 10 min. A 10% SDS-PAGE gel was prepared for electrophoresis, and 20 μg protein samples were loaded into each well. After transferring the protein samples to PVDF membranes, we first incubated the protein-containing PVDF membranes with 5% skim milk and then used anti-PANX1 (1:4000, 12595-1-AP, Proteintech, Wuhan, China) and anti-GAPDH (1:10,000, 60004-1-Ig, Proteintech, Wuhan, China), two primary antibodies, which were incubated on the membrane. Next, they were incubated with secondary antibodies (1:5000, BA1050, BA1054, Boster, Wuhan, China) for 2 h at room temperature. Finally, we used ECL reagents to perform protein expression assays.

### 2.15. Proliferation Evaluation

One of the methods was the use of Cell Counting Kit-8 (CCK-8) (MedChemExpress, Monmouth Junction, NJ, USA). At a density of 1 × 10^3^ cells per well, cells were plated in 96-well plates.

Once the cells had fully adhered to the wall, the medium in the culture wells was aspirated individually. Subsequently, CCK-8 working solution, prepared at a ratio of 1:9, was added to each well. The wells were then placed in the cell culture incubator and incubated in subdued light for 2 h. Following this, the OD value of each well was measured at 450 nm. Additionally, the OD values were recorded 24 h, 48 h, and 72 h after the cells had adhered to the wall. Subsequently, proliferation curves for the different groups of PANX1-expressing cells were plotted.

The other method was he use of the EdU Kit (Beyotime Institute of Biotechnology, Shanghai, China). A 200 μL measure of EdU solution was added to each well that 2 × 10^4^ cells were seeded onto before and incubated at a temperature of 37 °C for two hours. A 4% paraformaldehyde buffer was used to fix cells for 15 min at room temperature. To remove the fixative, the cells were washed three times in PBS buffer for five minutes. Membrane permeabilization with 0.5% Triton X-100 allowed the dye to enter the cells. This step took 20 min. Finally, cells were incubated with the dye Hoechst 33342 for 20 min in a light-protected environment after reacting with a click solution for 30 min. Fluorescence revealed the presence of EdU-positive cells.

### 2.16. Wound-Healing Assay

The capacity of cells to migrate was evaluated with the use of the wound-healing assay. We scraped the cells with the tip of a 200 μL pipette, rinsed them three times in phosphate buffer, and incubated them at 37 °C with serum-free media. We set two time points in the experiment, 0 h and 24 h. At each of these two time points, photographs were taken with an inverted microscope to obtain photographs of the state of the cells at the wounds.

### 2.17. Transwell Assay

The invasive potential of cells was measured using the following method. First, we put matrixgel and PBS (1:8) in the top chamber of a 24-well transwell plate (Corning, Corning, NY, USA) and incubated them in a sterile atmosphere until the mixture gelled. After that, 2 × 10^5^ cells were put into the top chamber and cultured with 100 μL of serum-free media, while 600 μL of medium containing 20% fetal bovine serum was added to the bottom chamber. After 48 h, we gently removed non-invasive cells from the top chamber using cotton balls and submerged the chambers in 4% methanol followed by 0.1% crystal violet. Finally, photographs were taken under an inverted microscope.

### 2.18. Statistical Analysis

The R software, version 4.2.2, was used throughout the course of the bioinformatics analysis. For the statistical analysis of molecular biology experiments, GraphPad Prism 8 was utilized. To analyze the differences in the data between the two groups, unpaired Student *t*-tests were carried out. In the case of three or more groups, the parametric and nonparametric One-Way ANOVA and Kruskal–Wallis tests were applied, respectively. For statistical significance, a value of *p* < 0.05 was considered to be meaningful.

## 3. Results

### 3.1. Identification of Genes with Differential Expression Associated with DAMPs

**Figure 2:**
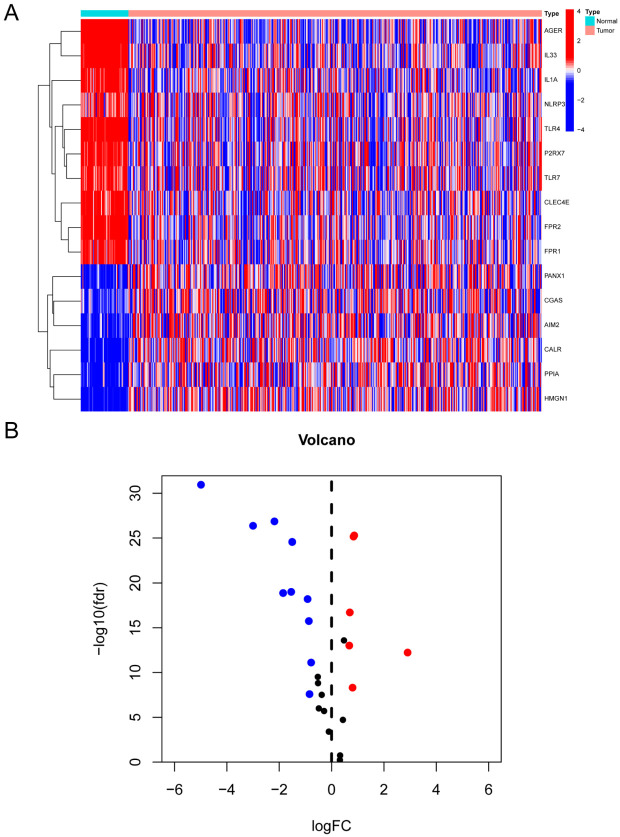
Identification of differentially expressed genes associated with DAMPs. Genes differentially expressed in normal tissues and LUAD associated with DAMP were shown in the heatmap (**A**) and volcano map (**B**).

To analyze the significance of ICD-relevant DAMPs and genes related to sensing receptors, we examined the levels of expression of 32 genes in LUAD samples taken from TCGA, comparing the levels of expression in 497 tumor samples to 54 non-tumor samples. Ten down-regulated genes (*AGER*, *IL33*, *IL1A*, *NLRP3*, *TLR4*, *P2RX7*, *TLR7*, *CLEC4E*, *FPR2*, *FPR1*) and six up-regulated genes (*PANX1*, *CGAS*, *AIM2*, *CALR*, *PPIA*, *HMGN1*) were considered differentially expressed in LUAD based on the selection criteria of a |logFC| ≥ 0.585 (variance multiplier > 1.5) and an FDR < 0.05 (Figure 2A,B).

### 3.2. Development and Verification of a DAMP-Based Risk Signature

**Figure 3:**
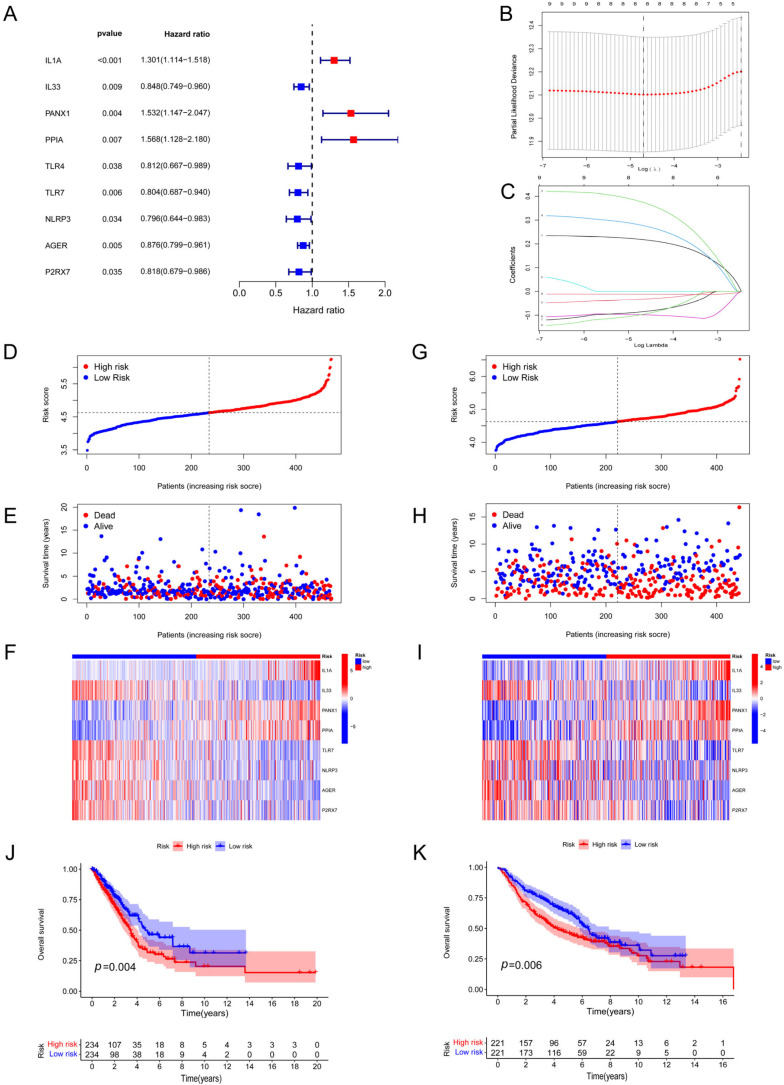
Construction and validation of a DAMP-based risk signature. (**A**) Univariate Cox analysis of DAMP-related prognostic DEGs. (**B**,**C**) Adjustment of coefficient profiles of 9 prognostic genes using LASSO regression analysis. (**D**–**F**) Risk score distribution (**D**), survival status of each patient (**E**), and heatmap of the prognostic 8-gene signature (**F**) in the TCGA database. (**G**–**I**) Risk score distribution (**G**), survival status of each patient (**H**), and heatmaps of the prognostic 8-gene signature (**I**) in the GEO database. (**J**,**K**) Kaplan–Meier analysis of the prognostic significance of the risk model in TCGA and GEO databases.

Nine DAMP-associated DEGs were related to LUAD patients’ OS, as determined using univariate Cox analysis (Figure 3A). Supplementary Figure S2 provides details on mutations in these nine genes. We successfully constructed a risk signature consisting of eight genes (*ILA*, *IL33*, *PANX1*, *PPIA*, *TLR7*, *NLRP3*, *AGER*, and *P2RX7*) based on nine candidate genes using LASSO regression analysis (Figure 3B,C). Risk score = (0.2185 × *IL1A*) + (−0.3324 × *IL33*) + (0.3760 × *PANX1*) + (0.2780 × *PPIA*) + (−0.0982 × *TLR7*) + (−0.0857 × *NLRP3*) + (−0.01185 × *AGER*) + (−0.0970 × *P2RX7*). Based on the above formula, the patient’s risk score can be calculated. The division of high-risk and low-risk groups was conducted due to the appropriate cutoff value of the risk score. According to the results of principal component analysis (PCA), all of the data may be effectively grouped into risk groups, as did the t-distributed stochastic neighbor embedding analysis (t-SNE) results (Supplementary Figure S3A–D). We also investigated the patterns of risk scores together with the status of survival and the expression of risk genes in TCGA (Figure 3D–F) and GEO (Figure 3G–I) datasets. In addition, the findings of our KM analysis of the two databases (Figure 3J,K) both revealed that the high-risk group had a poorer prognosis in terms of their expected outcomes.

### 3.3. The Risk Signature’s Correlation with the Clinical Characteristics

**Figure 4:**
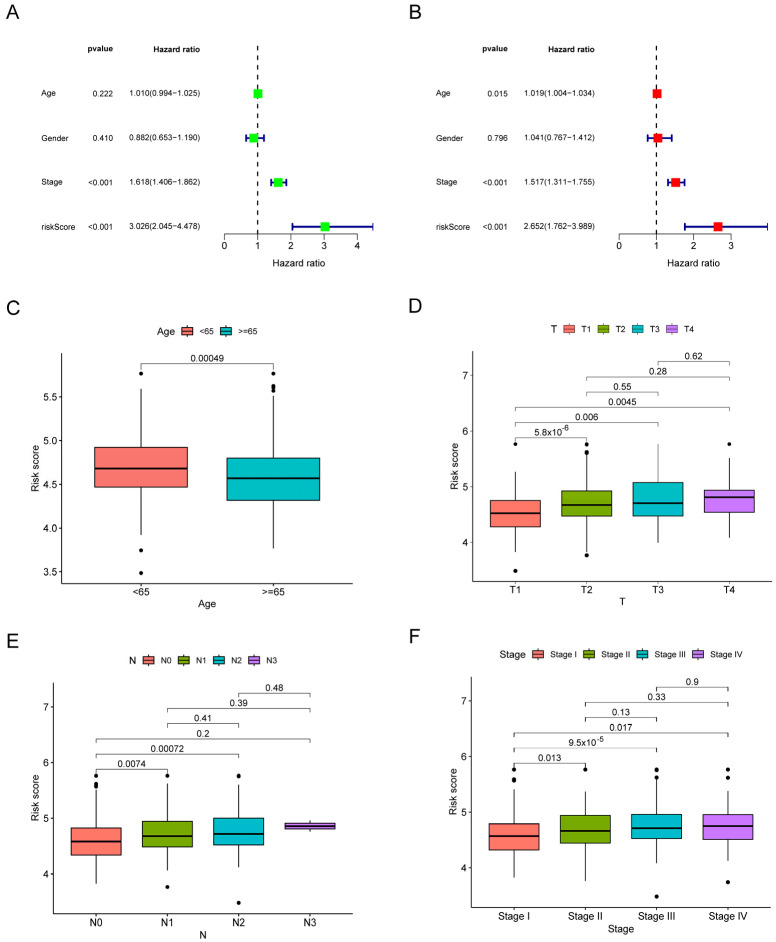
Correlation between the risk signature and clinical characteristics. (**A**) Univariate Cox analysis to assess the relationship between risk scores, clinical characteristics, and overall survival of patients with LUAD. (**B**) Multivariate Cox analysis to identify independent risk factors for overall survival in patients with LUAD. (**C**–**F**) Association of age (**C**), T stage (**D**), N stage (**E**), and pathological stage (**F**) with risk scores in patients with LUAD.

To better assess the risk signature’s prognostic value, we conducted Cox analyses, including univariate and multivariate analyses (Figure 4A,B). Results showed that the risk score might have prognostic capability on its own. Further, the analysis of the link between the risk score and numerous clinical factors provides further insight into the clinical value of the signature. As the results showed, several worse clinical features were highly related to increased risk scores (Figure 4D–F), and there was a difference in the signature between the two age categories (≥65 years or <65 years).

**Figure 5:**
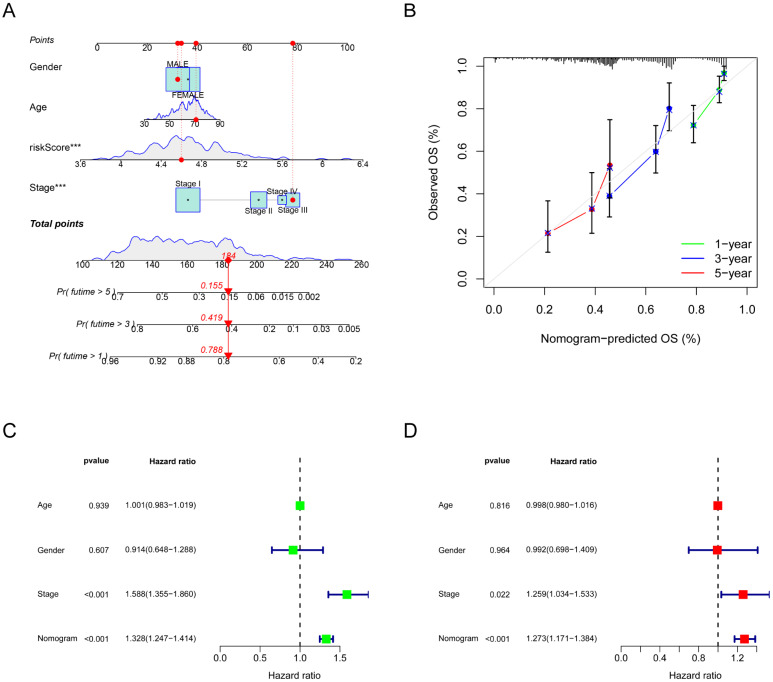
Constructed a nomogram combining the risk score and clinical characteristics. (**A**) Integration of clinical characteristics and risk scores to construct a nomogram to predict overall survival in patients with LUAD. (**B**) Calibration curves for the clinical prognostic nomogram. (**C**,**D**) Univariate and multivariate Cox analyses to assess the prognostic value of the nomogram. *** *p* < 0.001.

Figure 5A depicts a nomogram developed using the risk score and clinical characteristics. The nomogram’s ability to make accurate predictions of OS rates was confirmed by plotting calibration curves (Figure 5B), which showed a strong correlation between the predicted and observed rates of survival. For LUAD patients, the nomogram was found to be strongly related to the OS in Cox regression analyses, whereas other factors were not (Figure 5C,D), suggesting that the nomogram can more accurately predict patient prognosis and have great clinical application value.

### 3.4. Analysis of the Risk Groups Connected to Immunity

**Figure 6:**
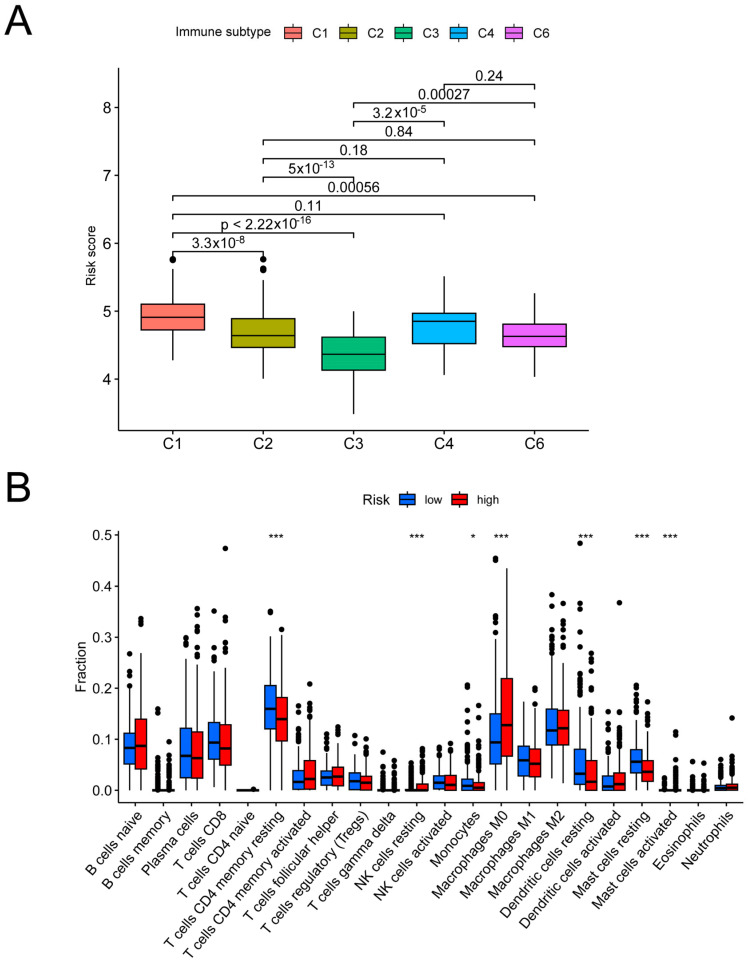

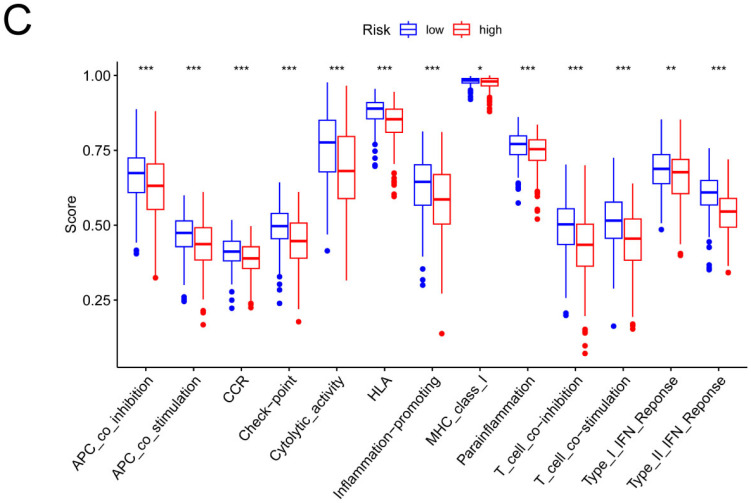
Differences in the immune features between the different risk groups. (**A**) Distribution of risk scores across the six different immune subtypes. (**B**) Differences in immune cell infiltration between the high- and low-risk groups. (**C**) Differences in immune function between the high- and low-risk groups. * *p* < 0.05, ** *p* < 0.01, *** *p* < 0.001.

First, in order to analyze the disparities in immunological characteristics that exist across the various risk groups, the correlation between risk scores and the molecular subtyping of immune phenotypes was studied. Based on immunogenomic analysis, immune subtypes were categorized into six types, C1–C6, with different subtypes having different immune-regulatory gene expression and prognosis [20]. And our results demonstrated that risk scores were substantially distinct across the C1-6 subtypes (Figure 6A), with C1 having the highest risk score and C3 the lowest. Following this, the TCGA dataset for LUAD patients was analyzed to compare the relative infiltration contents of immune cells (Figure 6B). In particular, resting CD4 memory T cells, monocytes, resting dendritic cells, and resting mast cells were positively connected with the low-risk group, but resting NK cells and activated mast cells were strongly correlated with the high-risk group. Apart from this, overall elevated immune functions of cell chemokine receptor (CCR) co-inhibition, checkpoint, and so on were shown in the group at a lower risk. In contrast, the opposite was found in the group at a higher risk (Figure 6C).

### 3.5. Analysis of the Sensitivity to Chemotherapeutic Drugs Based on Risk Groups

**Figure 7:**
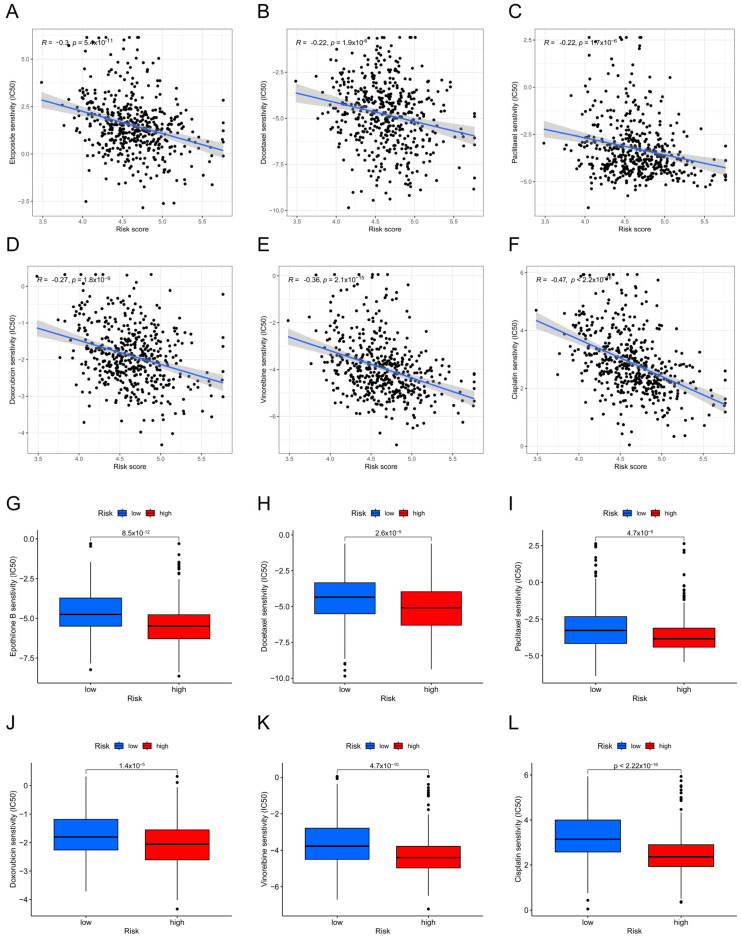
Differences in the chemotherapeutic drug sensitivity of different risk groups. (**A**–**F**) Correlation of sensitivity to Etoposide (**A**), docetaxel (**B**), paclitaxel (**C**), doxorubicin (**D**), vinorelbine (**E**), and cisplatin (**F**) with risk scores in patients with LUAD. (**G**–**L**) Differences in sensitivity to Etoposide (**G**), docetaxel (**H**), paclitaxel (**I**), doxorubicin (**J**), vinorelbine (**K**), and cisplatin (**L**) in the high- and low-risk groups of LUAD patients.

We estimated the IC50 values using the GDSC database and then used that information to make a prediction about the sensitivity of chemotherapeutic drugs to the two risk groups. Etoposide, docetaxel, doxorubicin, paclitaxel, vinorelbine, and cisplatin were all examples of conventional medications used to treat LUAD, and the findings indicated that their estimated IC50 values were all higher in the low-risk group (Figure 7A–L). This suggested that, compared to individuals with higher risk scores, the group at a lower risk had the tendency to be more resistant to chemotherapy.

### 3.6. Functional Enrichment Analysis

**Figure 8:**
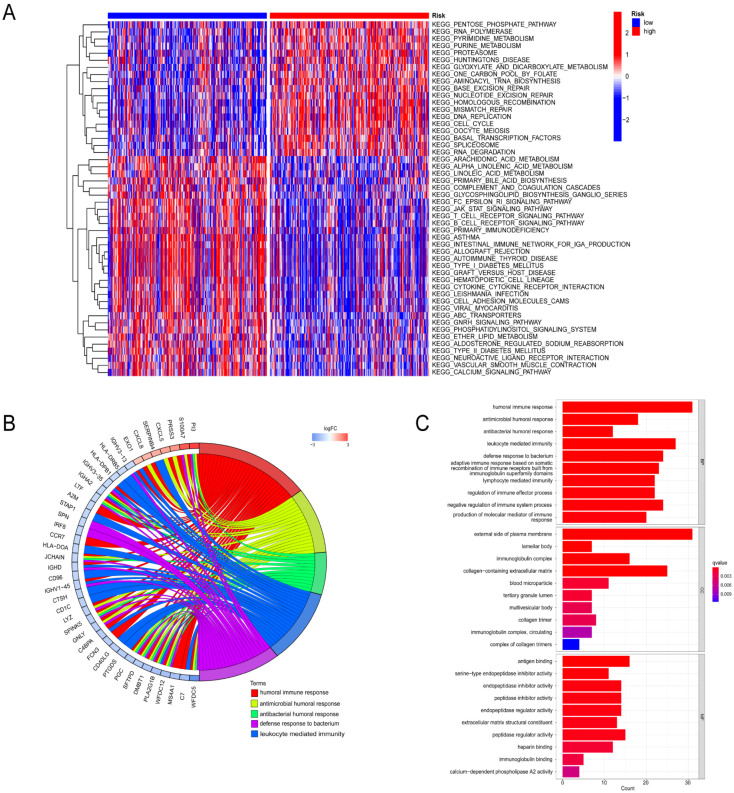

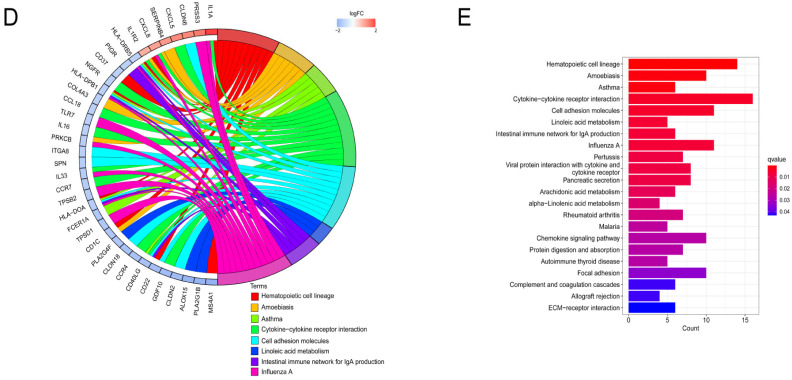
Enrichment analysis of the different risk groups. (**A**) Heatmap of GSVA analysis results in high- and low-risk groups. (**B**,**C**) GO analysis of DEGs in high- and low-risk groups. (**D**,**E**) KEGG analysis of DEGs in high- and low-risk groups.

With the aim of investigating the diverse functions of different risk groups, KEGG enrichment analysis was implemented together with GO enrichment analysis. In addition, utilizing GSVA enrichment analysis, we assessed the stimulation of biological signaling pathways within the two risk groups. Compared to the high-risk group, in the low-risk group, significant enrichment of the Fc epsilon RI, T, and B cell receptor signal pathways were observed (Figure 8A). Additionally, 391 DEGs were discovered between the two groups, and these DEGs were used for additional enrichment analyses. They were predominantly engaged with a variety of biological processes, such as humoral immune, antimicrobial humoral, antibacterial humoral, leukocyte-mediated immunity, and bacterial defense response, according to the findings of the GO enrichment analysis (Figure 8B,C). Furthermore, signaling pathways like hematopoietic cell lineage, amoebiasis, asthma, cytokine–cytokine receptor interaction, and cell-adhesion molecules were identified using KEGG enrichment analysis (Figure 8D,E).

### 3.7. Validation of the Hub Gene by WGCNA

**Figure 9:**
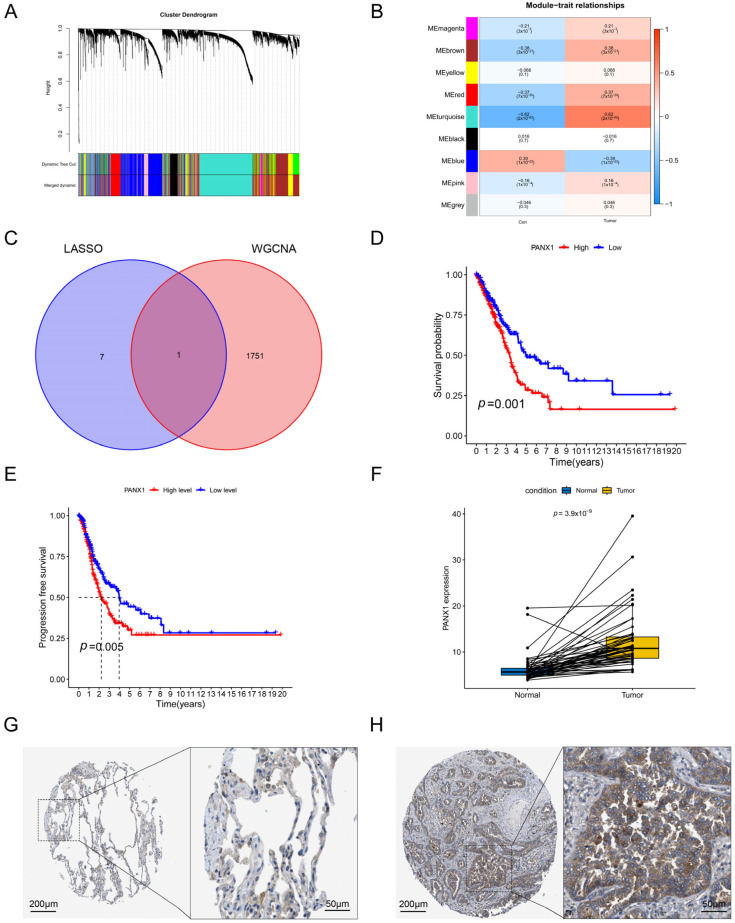
The diagnostic and prognostic value of PANX1 in LUAD. (**A**) Gene dendrogram of WGCNA. (**B**) Heatmap of the relationship between modules and LUAD traits. (**C**) Venn diagram of core module genes and genes involved in LASSO model taking the intersection. (**D**) Kaplan–Meier analysis of the prognostic significance of PANX1 in TCGA databases. (**E**) Progression-free survival analysis of PANX1 in high- and low-expression groups. (**F**) Paired analysis of PANX1 expression differences in normal and tumor tissues. (**G**,**H**) Immunohistochemical picture of PANX1 expression in normal (**G**) and tumor tissues (**H**) obtained from the HPA database.

**Figure 10:**
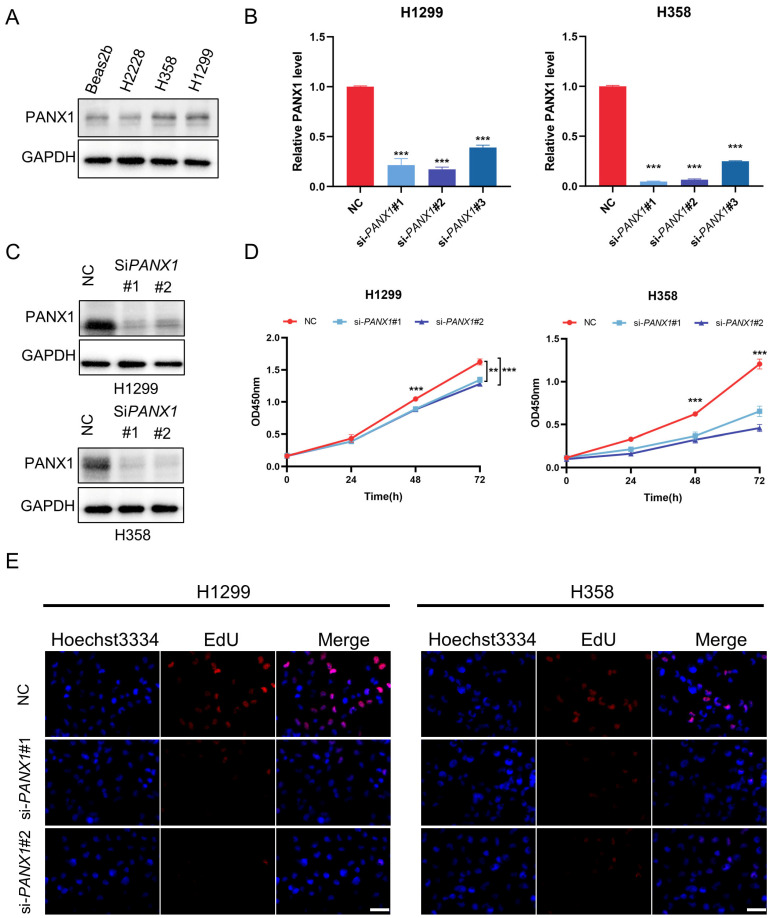

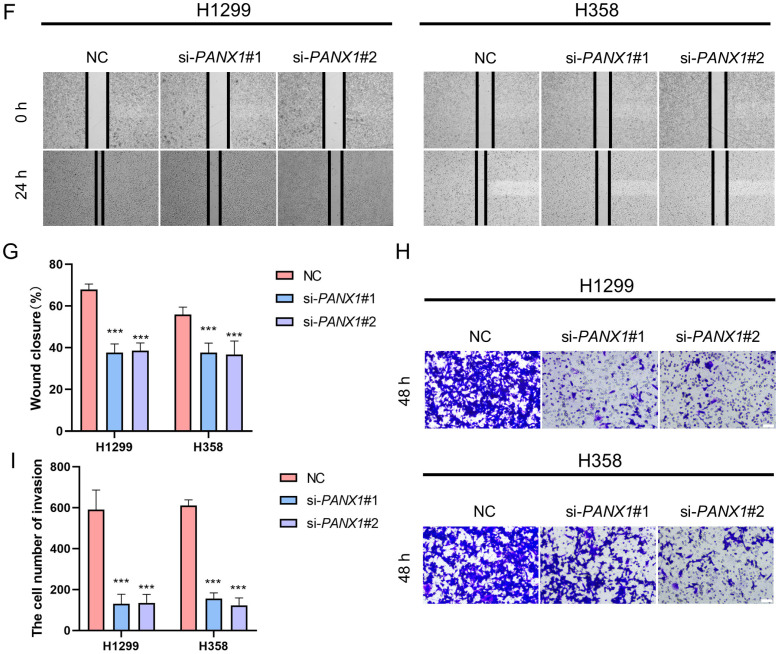
Experimental verification of the important role of PANX1 in lung adenocarcinoma. (**A**) Western blot analysis of PANX1 expression in lung adenocarcinoma cell lines and normal lung epithelial cells. (**B**) PCR confirmation of knockdown efficiency of *PANX1*. (**C**) Western blot analysis confirmation of knockdown efficiency of PANX1. (**D**) CCK-8 results. (**E**) EdU results (scale bar: 50 μm). (**F**) Wound-healing assay results. (**G**) Statistical analysis of the wound-healing assay results. (**H**) Transwell assay results (scale bar: 50 μm). (**I**) Statistical analysis of the transwell assay results. ** *p* < 0.01, *** *p* < 0.001.

We performed WGCNA analysis on the TCCA dataset, aiming to validate biomarkers of diagnosis and prognosis for LUAD. According to the module–trait association study, the turquoise module was exceptionally important (cor = 0.62, *p* = 2 × 10^−62^) (Figure 9A,B), and we took a cross-section of the genes in the grey module (1752) with the eight genes in the LASSO model (Figure 9C). Then, we identified a gene, *PANX1*, which has potential diagnostic and prognostic value in LUAD. We found that PANX1 was associated with overall survival in LUAD patients (Figure 9D). The analysis of the effect of PANX1 expression on progression-free survival (PFS) subsequently also revealed that PANX1 was closely associated with tumor progression (Figure 9E). PANX1 was shown to be overexpressed in tumor samples in the further investigation (Figure 9F). Results from the HPA database supported this finding (Figure 9G,H). This was further confirmed in Western blot experiments, which demonstrated that LUAD cell lines H358 and H1299 expressed PANX1 at considerably higher levels than normal lung epithelial cells BEAS-2B (Figure 10A).

### 3.8. Experimental Confirmation of the Crucial Function of PANX1 in Lung Adenocarcinoma

Given the above results indicating the high expression of PANX1 in H1299 and H358, we proceeded to investigate the pivotal role of PANX1 in LUAD by transfecting LUAD cell lines H1299 and H358 with siRNA-*PANX1* to downregulate the expression of PANX1. After confirming the effectiveness of the PANX1 knockdown with PCR and Western blot assays (Figure 10B,C), we found the findings of the Cell Counting Kit-8 and EdU tests demonstrated that the reduction in PANX1 decreased cell proliferation; meanwhile, cell invasion was inhibited, as determined by the wound-healing experiment and transwell test (Figure 10D–I). These experimental results indicated that PANX1 was a key molecule for tumor growth and metastasis, which may contribute to a worse prognosis for patients with lung adenocarcinoma.

### 3.9. Immune-Related Analysis of PANX1

**Figure 11:**
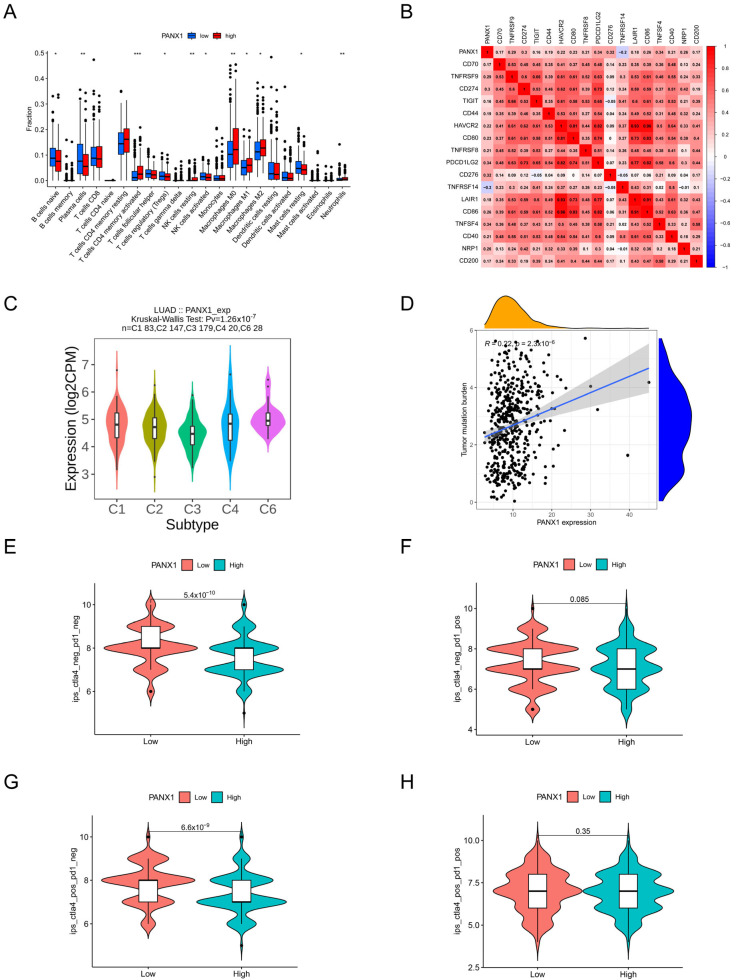
The relationship between PANX1 and immunity. (**A**) Relationship between PANX1 and immune cell infiltration. (**B**) Association between PANX1 and immune checkpoints. (**C**) Association between PANX1 and immune subtypes. (**D**) Relationship between PANX1 and TMB. (**E**–**H**) Relationship between PANX1 and TCIA immunotherapy. * *p* < 0.05, ** *p* < 0.01, *** *p* < 0.001.

According to further research into the connection between PANX1 and immunity, positive associations were discovered between PANX1 and naive B cells, T cells with CD4 memory activated, macrophages, and neutrophils (Figure 11A). Moreover, in terms of immune checkpoints, PANX1 and CD274, CD276 were positively correlated (Figure 11B) while having a negative association with *TNFRSF14*. The TISIDB website showed that in the C3 and C6 subtypes, respectively, PANX1 was expressed at the lowest and highest levels (Figure 11C). Furthermore, PANX1 expression and the tumor mutation burden (TMB) were in a positive relation (Figure 11D). In our study, we aimed to predict the responsiveness to immune checkpoint therapy in patients with high and low PANX1-expressing LUAD using the immunophenoscore (IPS). The IPS analysis included PD1 and CTLA4, which were further categorized into four sections: ips_ctla4_neg_pd1_neg (CTLA4-negative response and PD1-negative response), ips_ctla4_neg_pd1_pos (CTLA4-negative response and PD1-positive response), ips_ctla4_pos_pd1_neg (CTLA4-positive response and PD1-negative response), and ips_ctla4_pos_pd1_pos (CTLA4-positive response and PD1-positive response). Our results indicated that patients with low PANX1 expression exhibited higher ips_ctla4_neg_pd1_neg and ips_ctla4_pos_pd1_neg scores (Figure 11E–H). This suggests the hypothesis that patients with low PANX1 expression may have a better response to anti-CTLA-4 treatment alone.

**Figure 12:**
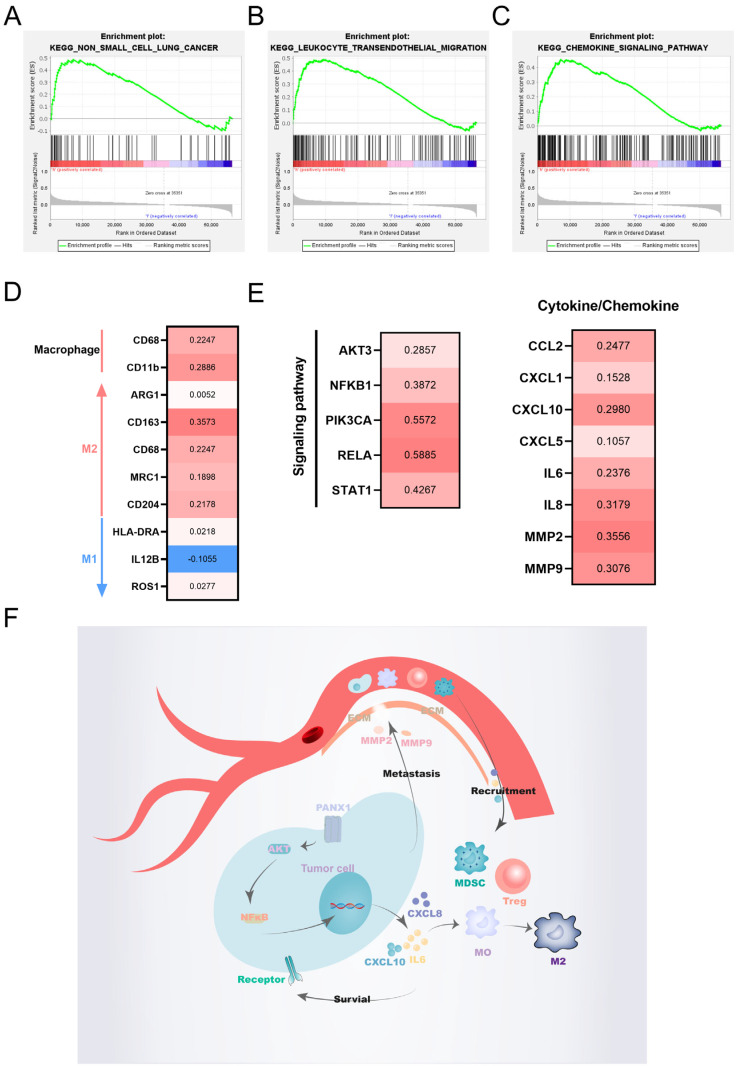
The regulation of the immune microenvironment by PANX1. (**A**–**C**) The results of GSEA analysis show that high expression of PANX1 is associated with non-small cell lung cancer (**A**), leukocyte transendothelial migration (**B**), and cytokine signaling pathways (**C**). (**D**,**E**) The correlation analysis results show the correlation between PANX1 with macrophage markers (**D**)and important molecules in cytokine pathways (**E**) using TIMER2.0. (**F**) Schematic representation of possible mechanisms by which PANX1 regulates the immune microenvironment.

Using GSEA analysis, PANX1 was found to be strongly associated with non-small cell lung cancer, leukocyte transendothelial migration, and cytokine signaling pathways (Figure 12A–C). Macrophages play an important role in the immune microenvironment, and further exploration of the relationship between PANX1 and macrophages revealed a significant positive correlation between PANX1 and M2 macrophage markers (Figure 12D). Further exploration of the possible causes revealed that PANX1 was positively correlated with important molecules in the cytokine pathway such as NFκB, AKT, and other factors such as IL6, CXCL8, and CXCL10, which are associated with inhibitory immune cell recruitment and macrophage polarization. In addition, PANX1 was closely associated with MMP2 and MMP9, which are factors associated with cell migration (Figure 12E). The relationship between PANX1 and the immune microenvironment is demonstrated in Figure 12F.

## 4. Discussion

Research on more effective therapies for lung adenocarcinoma has been ongoing for some time in response to the disease’s high death rate [21,22]. Immunotherapy for LUAD is an effective novel treatment modality, which includes the following approaches: PD1/PDL1 inhibitors, CTLA4 inhibitors, and CAR-T cell therapy [23,24,25,26]. PDL1 and CTLA-4 inhibitors are two of the current therapies for advanced NSCLC that have been approved by the FDA. However, immunotherapy benefits only a small percentage of patients in practical applications [27,28]. Immunobiomarkers play a crucial role in predicting the efficacy of immune checkpoint inhibitors. Common immune biomarkers currently include PD-L1 expression levels, TMB (tumor mutation burden), and TILs (tumor-infiltrating lymphocytes) [3,29,30]. PD-L1 expression levels are frequently used as predictors of the efficacy of immune checkpoint inhibitors, with higher PD-L1 expression levels typically associated with a more favorable therapeutic response. TMB, another important biomarker, has been shown to correlate with the efficacy of immune checkpoint inhibitors, and patients with high TMB may be more likely to benefit from immunotherapy. Additionally, measurements of TILs have been proposed to predict the efficacy of immunotherapy, with higher TIL levels generally linked to improved treatment responses.

However, these biomarkers are not infallible. For instance, due to PD-L1 expression levels being influenced by various factors, a clinical trial found that tumor PD-L1 status did not consistently predict patient response or survival after treatment with nivolumab [31]. Patients with elevated TIL levels may also experience disease progression following immunotherapy due to low PD-L1 expression or the presence of tumor margins only [32]. Moreover, there are patients with high mutation loads who do not respond to immune checkpoint inhibitors [33]. Expanding the benefits of immune checkpoint therapy will necessitate the discovery of new and effective immune markers.

The ineffectiveness of ICBs may be related to the tumor immunophenotype. “Cold tumors” do not respond well to immunotherapy because there are not enough immune cells around the edges and inside the tumor. In contrast, tumors with enough immune cells infiltrating tumor margins or interior are considered “hot tumors,” which respond well to immunotherapy [34,35]. Immunogenic cell death can help change the state of “cold tumors” to “hot tumors”, and immunogenic induction therapy in conjunction with immune checkpoint inhibitor therapy shows tremendous promise [13,14,36,37]. The release of DAMPs is the key to ICD, and accordingly, we hypothesize, for LUAD patients, that the expression of DAMP-associated genes would influence their prognosis and treatment and is expected to be a novel and effective marker for immunotherapy.

In the present study, we examined 32 DAMP-related genes in LUAD, found differentially expressed genes, and then selected 8 genes to construct an associated risk signature. The risk signature was further examined in connection to LUAD patients’ prognosis, clinical characteristics, drug sensitivity, and immunological microenvironment. In addition, enrichment analyses of DEGs in two risk groups were conducted. Finally, we confirmed that PANX1 expression substantially affects LUAD cell proliferation, and invasion capacity.

Two up-regulated genes, *PANX1* and *PPIA*, were ultimately included in the signature, along with six down-regulated genes: *ILA, IL33, TLR7, NLRP3*, *AGER,* and *P2RX7.* Patients with LUAD who were at a high risk for complications had notably poor overall survival, as shown by Kaplan–Meier survival curves, and these findings were corroborated by external validation using the GEO dataset. The analyses based on clinical characteristics demonstrated the clinical utility of the risk signature as well as the nomogram dependent on clinical characteristics and risk scores. Given that some chemotherapeutic drugs are identified as ICD inducers [38,39,40,41], we hypothesized that DAMP-related gene expression affects drug sensitivity in lung adenocarcinoma patients, and this study showed patients at a low risk were more resistant to these conventional drugs, suggesting that the risk signature guides clinical chemotherapy drug use.

The function of ICD is not solely determined by the antigenicity owing to dying cells, but adjuvant properties and the immune microenvironment are also critical. The spatially and temporally coordinated release or exposure of DAMPs gives them adjuvant properties and is needed for APCs to be recruited and mature [5,42]. Conditions in the immune microenvironment influence the initiation of adaptive immunity by immunogenic variant cells [43]. In a previous study, six immune subtypes were found in a study that characterized the immunological tumor microenvironment of 33 TCGA-analyzed tumors using an immunogenomic method, with C4 and C6 having the poorest prognosis compared to C3, which had a more favorable outcome [20]. In our study, the risk scores of C4 and C6 were high, while C3′s risk scores were low. In addition, it is noteworthy, in the group with low risk, that immune functions like T cell co-stimulation and immune checkpoints were activated, indicating that these patients responded better to ICB therapy compared to the other group.

Further analysis uncovered the fact that PANX1 has an extensive relationship with LUAD, which is a macropore protein that is present on many cells’ surfaces and allows several metabolites such as ATP to pass through it [44]. Many kinds of tumors are linked to abnormally high levels of PANX1 expression, including breast and skin tumors [45,46,47], but its study in lung adenocarcinoma is rare and deserves further investigation. In view of our results, we conclude that PANX1 influences the prognosis of LUAD patients. The role that PANX1 plays in immunotherapy is noticeable. In this study, we observed that anti-CTLA4 treatment alone may be more effective in the PANX1 low-expression group. Additionally, in contrast to the result that PANX1 releases ATP to promote immunogenic cell death, our study shows that, in lung adenocarcinoma, on the one hand, PANX1 may reduce immunogenic cell death by recruiting suppressor immune cells and promoting macrophage conversion to M2 type through cytokine pathways, and, on the other hand, these cytokines promote tumor cell survival and metastasis. These results indicate that PANX1 blockers paired with ICB may be a novel direction worth pursuing in tumor therapy.

In the present study, we are cognizant of the need to closely consider certain limitations. Risk-signature-predictive and guiding dose values have to be validated with a larger clinical sample, and biological experiments are needed to further investigate the role that PANX1 plays in immunity.

## 5. Conclusions

Overall, we developed a completely novel signature containing eight genes related to DAMPs, and it is capable of accurately assessing the prognosis of LUAD patients. This study furthers our understanding of DAMP-related genes’ importance in LUAD development and serves as a guide for immunotherapy and chemotherapy protocols for LUAD patients.

## Data Availability

The datasets used in this study can be found online as described above. The TCGA database is located at https://portal.gdc.cancer.gov, accessed on 3 December 2022, accession number: TCGA-LUAD; the GEO database is located at https://www.ncbi.nlm.nih.gov/gds, accessed on 3 December 2022, accession number: GSE68465.

## Supplementary Materials

The following supporting information can be downloaded at https://www.mdpi.com/article/10.3390/biom14010108/s1: Figure S1: The detailed analysis process of this study; Figure S2: Mutation information of 9 prognostic DAMP-related genes; Figure S3: PCA and t-SNE analysis of the signature; Figure S4: Original images for blots.

## Abbreviations

ICD: Immunogenic death; DAMPs: damage-associated molecular patterns; LUAD: lung adenocarcinoma; LASSO: the least absolute shrinkage and selection operator; OS: overall survival; WGCNA: weighted correlation network analysis; NSCLC: non-small cell lung cancer; ICBs: immune checkpoint inhibitors; APCs: antigen-presenting cells; ATP: adenosine-5′-triphosphate; CALR: calreticulin; TPM: transcripts per million; GEO: Gene Expression Omnibus; DEGs: differentially expressed genes; IC50: half maximal inhibitory concentration; IPS: immunophenotype scores; GSVA: gene set variation analysis; GO: Gene Ontology; KEGG: Kyoto Encyclopedia of Genes and Genomes; TOM: unsigned topological matrix; siRNA: small interfering RNA; WB: Western blot; CCK-8: Cell Counting Kit-8; CCR: cell chemokine receptor; TILs: tumor-infiltrating lymphocytes; TMB: tumor mutation burden.
