# Supplementary material for: Construction of a Novel Damage-Associated Molecular-Pattern-Related Signature to Assess Lung Adenocarcinoma’s Prognosis and Immune Landscape
Source: Biomolecules. 2024 Jan 15;14(1):108. doi: 10.3390/biom14010108 (PMC10813434; doi:10.3390/biom14010108)
Supplement: Supplementary file 1 [file biomolecules-14-00108-s001.zip › biomolecules-2714863-supplementary.pdf]

Supplementary Materials

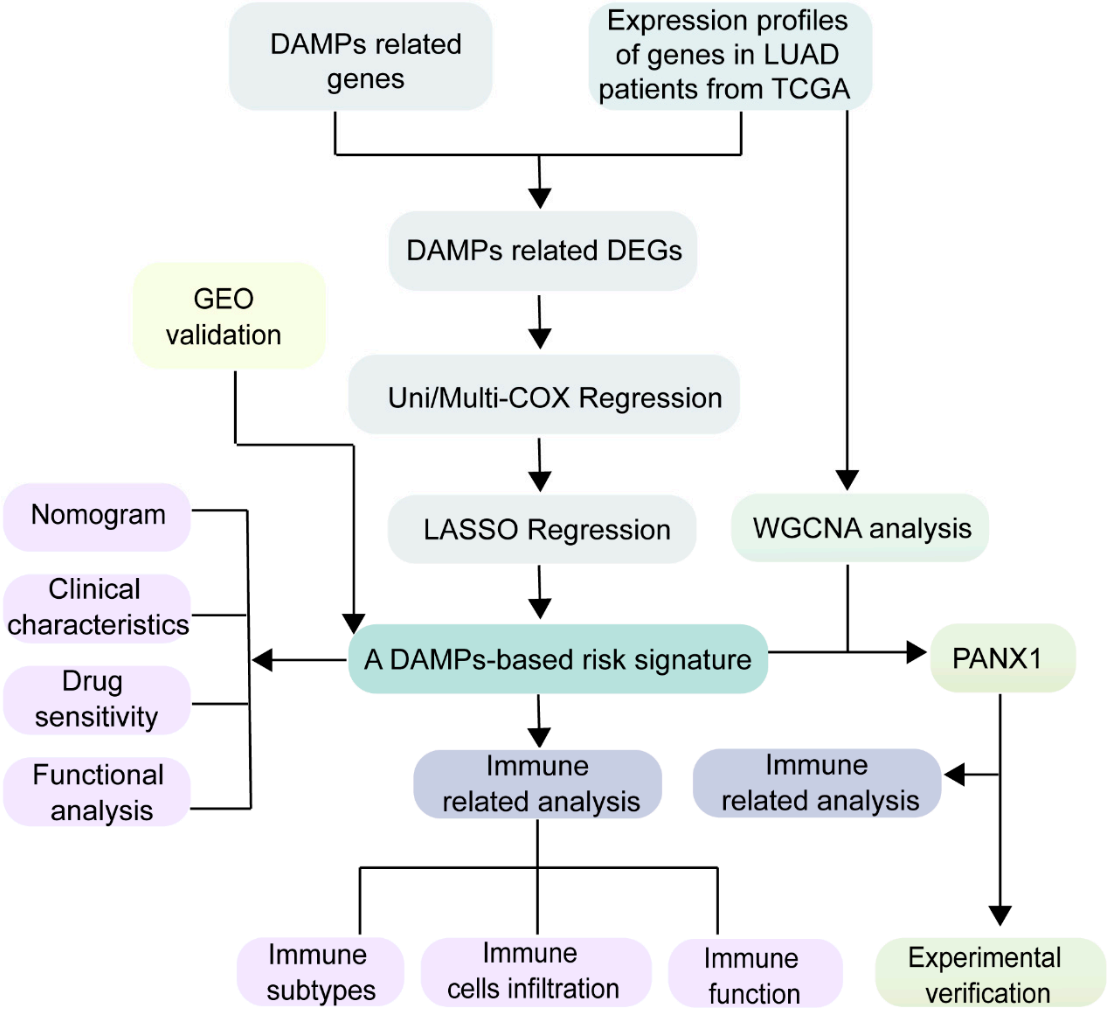

Figure S1. The detailed analysis process of this study.

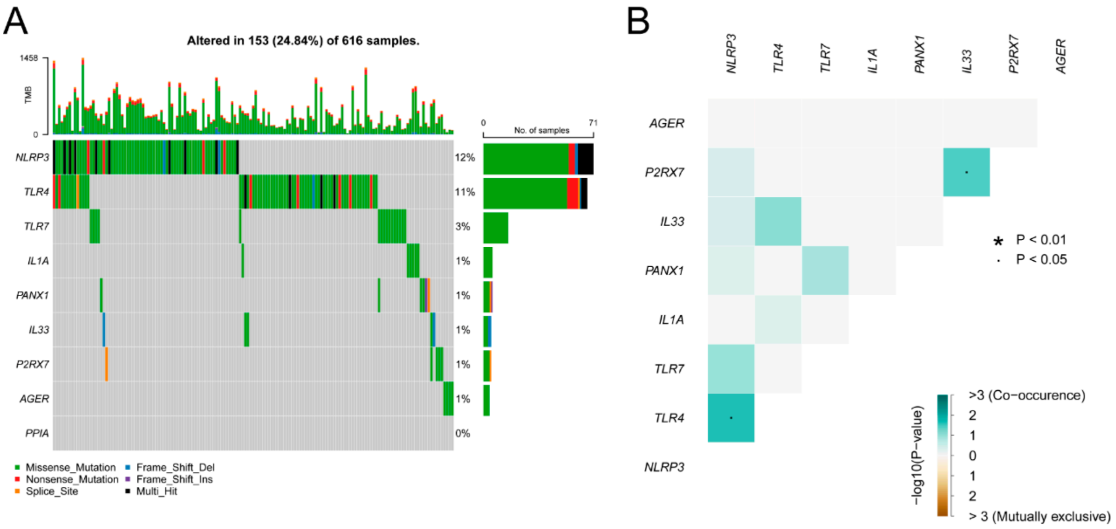

Figure S2. Mutation information of 9 prognostic DAMPs-related genes. **(A)** Waterfall plot of gene mutation types. **(B)** Gene co-mutations.

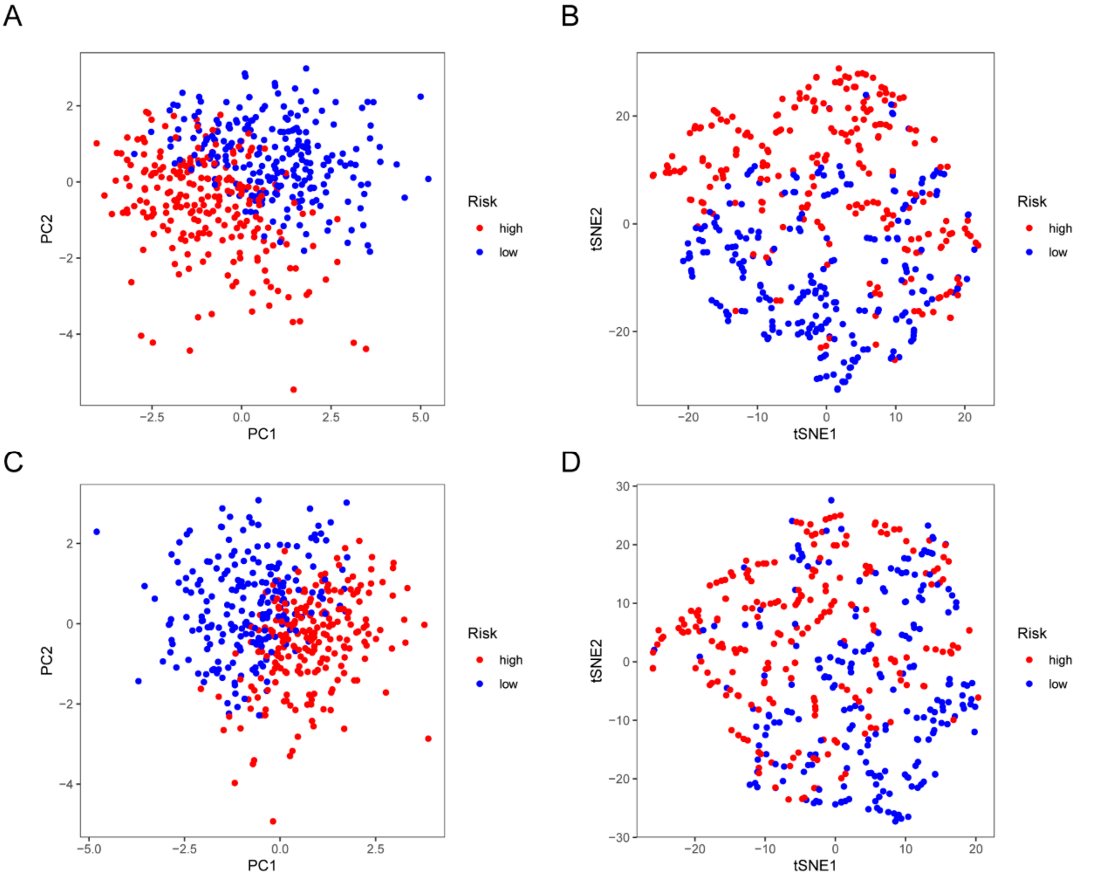

Figure S3. PCA and t-SNE analysis of the signature. **(A, B)** PCA (A) and t-SNE analysis (B) of TCGA database. **(C, D)** PCA (C) and t-SNE analysis (D) of GEO database.

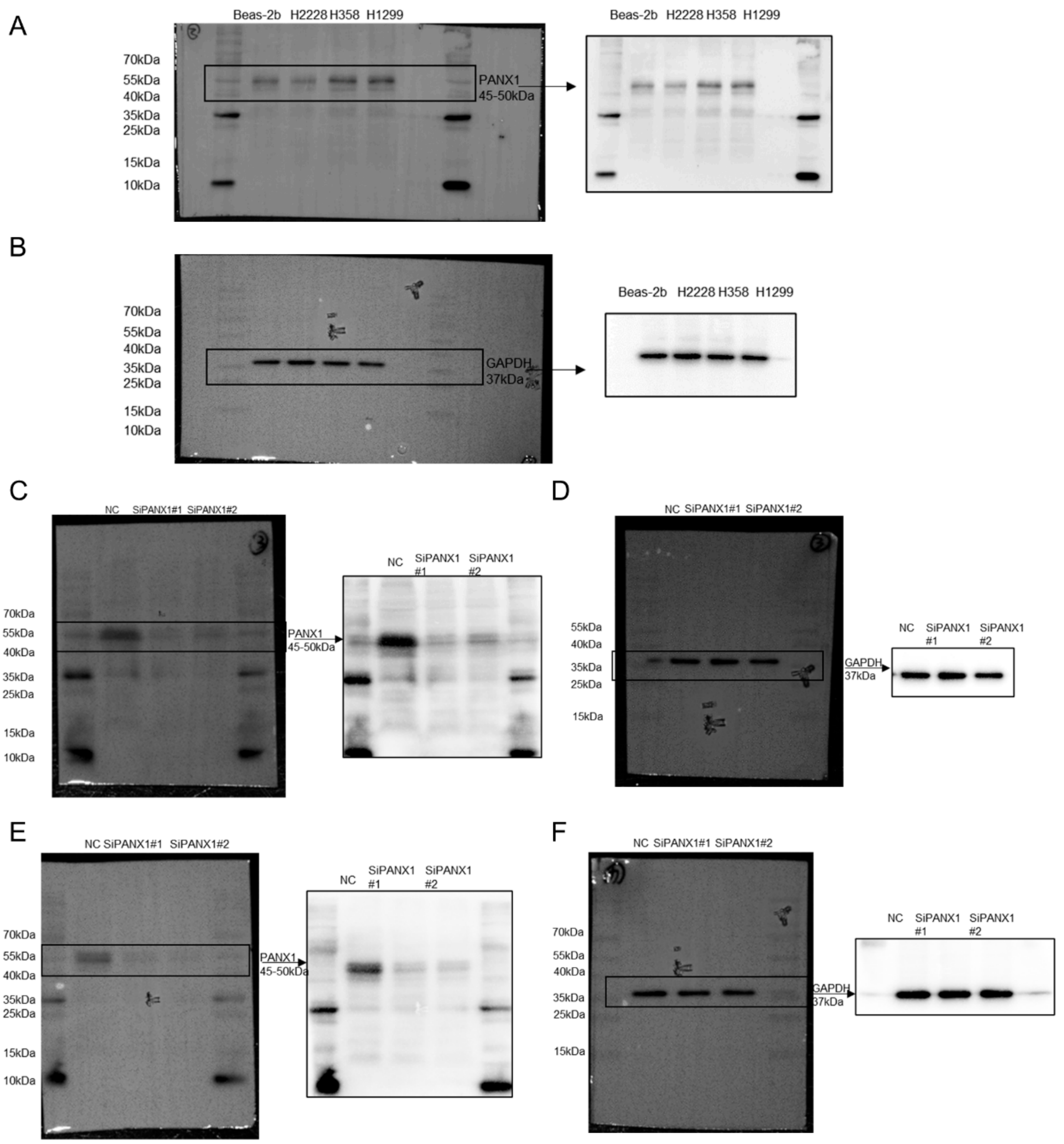

Figure S4. Original images for western blots. **(A)** Original western blot of PANX1 in Figure 10A. **(B)** Original western blot of GAPDH in Figure 10A. **(C)** Original western blot of H1299 PANX1 in Figure 10C. **(D)** Original western blot of H1299 GAPDH in Figure 10C. **(E)** Original western blot of H358 PANX1 in Figure 10C. **(F)** Original western blot of H358 GAPDH in Figure 10C.
